
JOURNAL INFORMATION
==============================
NLM Title Abbreviation: J Pharm Policy Pract
Journal ID: J Pharm Policy Pract
NLM Journal ID: 101627192

Journal of Pharmaceutical Policy and Practice

EISSN: 2052-3211
Publisher: Taylor & Francis

ARTICLE INFORMATION
==============================
PMCID: PMC12599563
DOI: 10.1080/20523211.2025.2579380
Article ID: 2579380
Article version: 1
Article version: Version of Record
Subjects: Abstract, Abstract

Abstracts from the 4th JoPPP-Thailand 2025 Conference

JOURNAL OF PHARMACEUTICAL POLICY AND PRACTICE

ABSTRACT

Electronic publication date: 2025 Nov 7
Publication date: 2025
Volume: 18
Issue: Suppl 3
Issue title: Abstracts from the 4th JoPPP-Thailand 2025 Conference
Electronic Location ID: 2579380
Copyright: © 2025 The Author(s). Published by Informa UK Limited, trading as Taylor & Francis Group
Copyright year: 2025
Copyright holder: The Author(s)
License: This is an Open Access article distributed under the terms of the Creative Commons Attribution License (http://creativecommons.org/licenses/by/4.0/), which permits unrestricted use, distribution, and reproduction in any medium, provided the original work is properly cited. The terms on which this article has been published allow the posting of the Accepted Manuscript in a repository by the author(s) or with their consent.
License URL: https://creativecommons.org/licenses/by/4.0/

Figure count: 0
Table count: 0
Equation count: 0
Reference count: 0
Page count: 39

==============================


Pharmacogenetic influence on tacrolimus therapy: impact of CYP3A5 and ABCC2 polymorphisms on graft survival and direct cost in kidney transplant recipients

Choong Chiau Ling 1
Islahudin Farida 1
Wong Hin-Seng 2 3
Yahya Rosnawati 4
Tahir Nor Asyikin Mohd 1
Makmor-Bakry Mohd 1
1 Center of Quality Medicine Management, Faculty of Pharmacy, Universiti Kebangsaan Malaysia , Jalan Raja Muda Abdul Aziz , 50300 , Kuala Lumpur , Malaysia
2 Department of Nephrology, Selayang Hospital , 68100 , Batu Caves , Selangor , Ministry of Health Malaysia
3 Sunway Medical Centre , Jalan Lagoon Selatan, Bandar Sunway , 46150 , Petaling Jaya , Selangor , Malaysia
4 Department of Nephrology, Kuala Lumpur Hospital , 50586 , Kuala Lumpur , Ministry of Health Malaysia
Corresponding author email: faridaislahudin@ukm.edu.my
Keywords: ABCC2, CYP3A5, kidney transplant recipients, polymorphism, clinical outcome, cost-effectiveness

Exploring the influence of complementary therapies on antiepileptic drug adherence: a sociodemographic analysis of Malaysian epilepsy patients

Ibrahim Umar Idris 1
Mohd Noor Siti Nor Aqilah 1
Jamshed Shazia 2
Long Chiau Ming 3
Abdul Jamil Ahmad Kamal Ariffin 1
Ahmad Nurulumi 1
Jamil Aslinda 1
Lim Kheng Seang 4
Ibrahim Khairul Azmi 5
Husin Mazlina 5
Sidek Norsima Nazifah 5
Basiam Sulaila 6
Daud Saidatul Manera Mohd 7
Lua Pei Lin 1
1 Faculty of Pharmacy, Universiti Sultan Zainal Abidin (UniSZA) , Kampus Besut, 22200 , Besut , Terengganu , Malaysia
2 School of Pharmacy, International Medical University (IMU) , Bukit Jalil, 57000 , Kuala Lumpur , Malaysia
3 School of Medical and Life Sciences, Sunway University , Bandar Sunway, 47500 , Selangor Darul Ehsan , Malaysia
4 Faculty of Medicine, University of Malaya , Lembah Pantai, Wilayah Persekutuan, 50603 , Kuala Lumpur , Malaysia
5 Hospital Sultanah Nur Zahirah , Jalan Sultan Mahmud, 20400 , Kuala Terengganu , Terengganu , Malaysia
6 Hospital Tengku Ampuan Afzan , Jalan Tanah Putih, 25100 , Kuantan , Pahang , Malaysia
7 Hospital Raja Perempuan Zainab II , Bandar Kota Bahru , Malaysia
Corresponding author email: umaribrahim@unisza.edu.my
Keywords: epilepsy, complementary and alternative medicine, medication adherence, sociodemographic factors, Malaysia, CAT, AED

Investigation of pharmacokinetic variation of voriconazole among cancer patients: a population pharmacokinetic study

Usman Muhammad 1
Akbar Zunaira 2
1 Institute of Pharmaceutical Sciences, University of Veterinary and Animal Sciences , Lahore , Pakistan
2 Riphah Institute of Pharmaceutical Sciences, Riphah International University , Lahore , Pakistan
Corresponding author email: usman.ips@uvas.edu.pk
Keywords: voriconazole, pharmacokinetics, neoplasms, therapeutic drug monitoring

Navigating usability in Clinical Decision Support System: a review of practices

Moktar Norlizawati 1
Wahab Mohd Shahezwan Abd 2
Zulkifly Hanis Hanum binti 2
Kasim Sazzli Shahlan bin 2
1 Ministry of Defence , Malaysia
2 University of Technology MARA , Malaysia
Corresponding author email: liza_moktar@yahoo.com
Keywords: clinical decision support systems, standards usability testing, user-computer interface, health information technology, evidence-based practice

Beyond the individual: a social ecological perspective on sustained self-monitoring blood glucose adherence and insulin dose self-adjustment among patients with diabetes in primary care

Salamat Nur Aini
Zulkifli Nur Wahida
Yen Wong Yuet
Karuppannan Mahmathi
Wahab Mohd Shahezwan Abd
Universiti Teknologi Mara (UiTM) , Malaysia
Corresponding author email: wahidazulkifli@uitm.edu.my
Keywords: Self-monitoring blood glucose, type 2 diabetes mellitus, adherence, qualitative study, Social Ecological Model

Assessment of sterile compounding practices: a descriptive observation in the PICU of a referral hospital

Genatrika Erza
Utaminingrum Wahyu
Namira Aura Sesi
Pharmacy Faculty, Universitas Muhammadiyah Purwokerto , Indonesia
Corresponding author email: erzagenatrika@gmail.com
Keywords: pharmaceutical preparations, drug compounding, sterility assurance, intensive care units, pediatric, microbial contamination

Identifying risk of hospital-acquired infections caused by multidrug-resistant organisms at critical care setting in UAE

Jumana Alsalloum 1 2
Noor Harun Sabariah 1
Subish Palaian 3
1 Univerisiti Sains Malaysia , Malaysia
2 University of Sharjah , United Arab Emirates
3 Ajman University , United Arab Emirates
Corresponding author email: sabariahnoor@usm.my
Keywords: drug resistance, bacterial, cross infection, risk factors, intensive care units

Evaluation of vitamin D deficiency in relation to autoimmune diseases among hospitalised patients at Central Jakarta Hospital

Fitriani 1
Ramatillah Diana Laila 1
Luthfiana Farisa 1
Dyanto Norman 1
Khan Kashif Ullah 2
Husna Ihsanil 3
Muchtiar Mulyadi 4
Darsono Michelle 1
Bur Rika 4
1 Department of Clinical Pharmacy, Faculty of Pharmacy, Universitas 17 Agustus 1945 Jakarta , Jakarta, Indonesia
2 Department of Clinical Pharmacy and Pharmacy Practice, Faculty of Pharmacy, University Malaya , Malaysia
3 Department of Internal Medicine, Islamic Hospital Jakarta Cempaka Putih , Jakarta , Indonesia
4 Department of Internal Medicine, YARSI Hospital , Jakarta , Indonesia
Corresponding author email: diana.ramatillah@uta45jakarta.ac.id
Keywords: vitamin D deficiency, autoimmune diseases, cross-sectional study, immunomodulation, Jakarta

Sarcopenia prevalence and its association with medication-related factors among geriatric outpatients at a tertiary hospital in Malaysia: a cross-sectional study

Hisham Siti Aishah Ikmal 1
Aziz Siti Azdiah Abdul 2
Zainal Zainol Akbar 2
Embong Julaida 1
Zhi Hor Pei 1
1 Hospital Kuala Lumpur , Malaysia
2 Universiti Kebangsaan Malaysia , Malaysia
Corresponding author email: sitiaishah.ih@gmail.com
Keywords: sarcopenia, aged, polypharmacy, muscle strength, adverse reactions

Identification and factor-associated analysis of potentially inappropriate medications (PIMs) based on STOPP START criteria in geriatric patients with kidney diseases at Undata Hospital, Palu, Indonesia

Safarudin Rudi 1 2
Annisa Arni Aplimeiraty 1
Faustine Ingrid 1
Adisaputra Arya Dibyo 1
Susilawati Ni Made 1
Hardi Hardi 1
Kusumawaty Afriani 1
Hardani Muhammad Fakhrul 1
1 Department of Pharmacy, Faculty of Mathematics and Natural Sciences, Tadulako University , Palu , Central Sulawesi , Indonesia
2 The Prescription Drug Misuse Education and Research (PREMIER) Center, University of Houston, College of Pharmacy , Houston , Texas , USA
Corresponding author email: rudi17safarudin@gmail.com
Keywords: potentially inappropriate medications, STOPP START criteria, geriatric patients, kidney diseases, medication safety, Palu

Side effects of using antihypertensive drug in hypertension patients: a systematic review of literature

Oktariana Elta 1
Ramatillah Diana Laila 1
Shehab Naglaa Gamil 2
1 Universty 17 Agustus 1945 Jakarta , Jakarta, Indonesia
2 Dubai Medical University , United Arab Emirates
Corresponding author email: eltaoktariana31@gmail.com
Keywords: side effect, hypertension, drugs, antihypertensive

Contribution factors of hypertension among adolescents: systematic literature review and bibliometric analysis

Rizkiya Ainur 1
Ramatillah Diana Laila 1
Khan Kashif Ullah 2
1 Pharmacy Faculty, Universitas 17 Agustus 45 Jakarta, Jakarta , Indonesia
2 Department of Clinical Pharmacy and Pharmacy Practice, Faculty of Pharmacy, Universiti Malaya , Kuala Lumpur , Malaysia
Corresponding author email: ainur.rizkiya@gmail.com
Keywords: hypertension, adolescents, blood pressure, obesity, systolic blood pressure, body mass index

Effectiveness of tuberculosis medication services in health care facilities: a systematic review of literature

Tumangger Safaruddin
Sormin Ida Paulina
Study Program of Pharmacy, Faculty of Pharmacy, Universitas 17 Agustus 1945 Jakarta , Jakarta 14350 , Indonesia
Corresponding author email: safaruddintgr@gmail.com
Keywords: tuberculosis, medication adherence, delivery of health care, treatment outcome

Structural Equation Modelling of the mediating role of social-environmental factors in enhancing Rational Drug Use Literacy among Village Health Volunteers in Thailand

Sakpirom Thunpitcha 1
Maluangnon Kusawadee 1
Sooksriwong Cha-oncin 1
Ingard Apinya 2
1 Faculty of Pharmacy, Thammasat University , Thailand
2 Faculty of Information and Communication Technology, Silpakorn University , Thailand
Corresponding author email: kusawade@tu.ac.th
Keywords: Rational Drug Use Literacy, Village Health Volunteers, social-environmental factors, situational factors, Structural Equation Modelling

The impact of domestic component level (TKDN) policy on medicine procurement: a regulatory perspective from Indonesia

Pratita Rasta Naya
Rahmawati Ayu
Manninda Reise
Anggriani Yusi
Universitas Pancasila , Indonesia
Corresponding author email: rastanayapratita@univpancasila.ac.id
Keywords: drug procurement, health policy, drug costs, pharmaceutical preparations, distribution

Analysis of anti-diabetic drug procurement prices with government reference prices

Sooksriwong Cha-oncin 1 2
Maluangnon Kusawadee 1 2
Hatpatoom Nattarat 1
Warasinchai Pitchayapa 1
Wongseree Pimchanok 1
1 Faculty of Pharmacy, Thammasat University
2 Drug Information and Consumer Protection Center (DICPC) , Thammasat University
Corresponding author email: kusawade@tu.ac.th
Keywords: drug procurement price, median price, reimbursement price

Outpatient reimbursement policies for cancer treatment in China: a comparative and longitudinal analysis

Wei Haoqi
Zhang Jinwei
Liu Xingchen
Liu Xingyu
Liu Xiaoyong
Hu Shuchen
Yan Kaining
Yang Caijun
Xi’an Jiaotong University , China
Corresponding author email: yangcj@xjtu.edu.cn
Keywords: outpatient reimbursement, medical insurance, cancer, policy recommendation

Pharmacy Policy 5.0: strategizing smart regulation to integrate AI and digital health international pharmaceutical systems

Dau Leonesia
Ramatilah Diana Laila
Pharmacy Faculty, Universitas 17 Agustus 1945 Jakarta , Jakarta 14350 , Indonesia
Corresponding author email: leonesiadau92@gmail.com
Keywords: Pharmacy Policy 5.0, intelligent regulation, AI in pharmacy, digital health, ASEAN, systematic review

Review of the implementation of the common illness care policy in pharmacy under the Universal Health Coverage scheme in Thailand

Uansri Sonvanee 1
Phodha Tuangrat 2
Maluangnon Kusawadee 2
Singweratham Noppcha 3
Babar Zaheer Ud-din 4
1 Faculty of Pharmacy, Thammasat University , Rangsit Campus , Pathum Thani , Thailand
2 Drug Information and Consumer Protection Center, Center of Excellence in Pharmacy Practice and Management Research, Faculty of Pharmacy, Thammasat University , Rangsit Campus , Pathum Thani , Thailand ; 99 Paholyothin Road, Khlong Nueng , Khlong Luang District , Thailand
3 Faculty of Public Health, Chiang Mai University , 239, Huay Kaew Road, Muang District , Chiang Mai , 50200 , Thailand
4 Department of Pharmacy, University of Huddersfield , UK ; University of Huddersfield , Queensgate, Huddersfield HD1 3DH , UK
Corresponding author email: tuangrat25@tu.ac.th
Keywords: common illness care, community pharmacy, Universal Health Coverage, health policy implementation, monitoring and evaluation, Thailand

Cost-effectiveness analysis of colonoscopy for colorectal cancer screening: a systematic literature review

Suardiana I Kadek 1
Kurnianta Putu Dian Marani 1
Candraningrat I Dewa Agung Ayu Diva 2
Amin Muhammad Qowiyul 3
Phodha Tuangrat 4
1 Bachelor of Pharmacy Study Program, Faculty of Pharmacy and Health Sciences, Universitas Pendidikan Nasional , Indonesia
2 Department of Pharmacology and Clinical Pharmacy, Faculty of Pharmacy, Mahasaraswati University , Denpasar , Bali , Indonesia
3 Faculty of Pharmacy, Universitas Gadjah Mada , Yogyakarta , Indonesia
4 Drug Information and Consumer Protection Center, Faculty of Pharmacy, Thammasat University , Rangsit Campus , Pathum Thani , Thailand ; Center of Excellence in Pharmacy Practice and Management Research Unit, Faculty of Pharmacy, Thammasat University , Rangsit , Thailand
Corresponding author email: kadeksuardiana@undiknas.ac.id
Keywords: colonoscopy, colorectal cancer, screening, cost-effectiveness, systematic review

Workforce distribution and retention: a pharmacist's perspective on deployment strategies after relocating from West to East Malaysia

Gill Manvikram Singh
Ministry of Defence , Malaysia
Corresponding author email: manvikramsinghgill@rocketmail.com
Keywords: workforce distribution, pharmacist retention, rural healthcare, East Malaysia

Improving ART adherence through the MIND module: validation and pilot testing among people living with HIV in Malaysia

Rizal Mohemmad Redzuan Mohemmad 1
Zaini Syahrir 1
Rahman Norny Syafinaz Ab 1
Musa Ramli 2
1 Kulliyyah of Pharmacy, International Islamic University of Malaysia , Malaysia
2 Kulliyyah of Medicine, International Islamic University of Malaysia , Malaysia
Corresponding author email: redzuanrizal@moh.gov.my
Keywords: HIV, antiretroviral therapy, medication adherence, psychosocial support, MIND Module, pilot testing

Assessment of knowledge, attitude, practices, and perceptions of first-year university students and university employees about antimicrobial stewardship: a quasi-intervention study

Idulsa Elsen
Urbiztondo Keint Erm
Anobling Bernadette D.
De Vera-Barasi Estela
Cariaga Maria Fay Nenette
Ocampo Justine Marie
University of Makati , Philippines
Corresponding author email: eidulsa.a12137468@umak.edu.ph
Keywords: antimicrobial stewardship, drug resistance, microbial health knowledge, attitudes, practice, pharmacy

Cost-utility analysis of FISH and RT-PCR testing followed by imatinib or corticosteroid treatment for hypereosinophilic syndrome in Thailand

Saramunee Kritsanee 1
Phimarn Wiraphol 1
Leelathanalerk Areerut 1
Chanasophon Suratchada 1
Srimongkon Pornchnok 1
Paktipat Pawich 1
Chantaphakul Hiroshi 2
Laisuan Wannada 3
Chantrathannachart Pichika 3
1 Faculty of Pharmacy, Mahasarakham University
2 Faculty of Medicine, Chulalongkorn University
3 Faculty of Medicine Ramathibodi Hospital, Mahidol University
Corresponding author email: kritsanee.s@msu.ac.th
Keywords: hypereosinophilic syndrome, FISH, RT–PCR, steroid, imatinib, cost-utility

Strengthening clinical pharmacy services through policy reform: the results from a comprehensive literature review

Salsabila Dafitri
Hutabarat Rahmi
University of 17 Agustus 1945 Jakarta , Jakarta, Indonesia
Corresponding author email: dafitrisalsabila00@gmail.com
Keywords: clinical pharmacy, policy reform, medication safety, therapeutic monitoring, health financing, patient-centered care

Improving maternal knowledge of vitamin D and stunting prevention: a pre-post study in urban Indonesia

Ramatillah Diana Laila 1
Luthfiana Farisa 1
Prima Sylvia Rizky 1
Susilowati Sri Endah 1
Khan Khasif Ullah 2
Darsono Michelle 1
Fitriani 1
Lestari Mega 1
Ghassani Fatiha Azka 1
1 University of 17 Agustus 1945 , Jakarta , Jakarta, Indonesia
2 University Malaya , Malaysia
Corresponding author email: farisa.luthfiana@uta45jakarta.ac.id
Keywords: Vitamin D deficiency, maternal health education, pregnant, child development disorders, nutritional

Detection of Staphylococcus aureus and determination of antibiotic resistance in asymptomatic carriers

Sambuu-yondon Idertungalag
Baljinnyam Enkhgerel
Dorjgochoo Amgalanzaya
Samdan Gaadulam
Erdene Enkhtaivan
Etugen University , Mongolia
Corresponding author email: enkhgerel.b@etugen.edu.mn
Keywords: Staphylococcus aureus, methicillin-resistant Staphylococcus aureus, drug resistance, bacterial, nasal cavity

Prevalence, motivations, usage patterns, and sociodemographic predictors of herbal medicine use among COVID-19 patients in Qatar

Nazar Zachariah 1
Shaito Abdullah 2 3 4
Alsheikh Raneem 4
Alsharif Fatima R. 4
Alwisi Nouran 4
Shi Zumen 5
1 College of Pharmacy, QU Health, Qatar University , Qatar
2 Biomedical Research Center, QU Health, Qatar University , Qatar
3 Department of Biomedical Sciences, College of Health Sciences, QU Health, Qatar University , Qatar
4 College of Medicine, QU Health, Qatar University , Qatar
5 Department of Human Nutrition, College of Health Sciences, QU Health , Qatar
Corresponding author email: znazar@qu.edu.qa
Keywords: COVID-19, medicine, traditional herbal medicine, complementary therapies

Assessment of drug-drug interactions in people living with HIV on antiretroviral and anti-cancer therapy

Radhakrishnan Rajesh 1
Raghuram Rao Nishika 1
Chandana Valipay Hari 1
Varma Danturulu Muralidhar 2
Pai Ananth 3
1 Department of Pharmacy Practice, Manipal College of Pharmaceutical Sciences, Manipal Academy of Higher Education , Manipal – 576 104 , Karnataka , India
2 Department of Infectious Diseases, Kasturba Medical College, Manipal Academy of Higher Education , Manipal – 576 104 , Karnataka , India
3 Department of Medical Oncology, Kasturba Medical College, Manipal Academy of Higher Education , Manipal – 576 104 , Karnataka , India
Corresponding author email: rajesh.r@manipal.edu haricv2701@gmail.com
Keywords: antiretroviral therapy, anti-cancer therapy, drug–drug interactions

More than a device: mothers’ perspectives on the life-changing impact of insulin pumps in children with diabetes in the United Arab Emirates

Fattah Lean Abdul 1
Mustafa Nour 1
Farajallah Alaa 2
Shankar Pathiyil Ravi 3
Palaian Subish 2
1 College of Pharmacy and Health Sciences, Ajman University , Ajman , UAE
2 Department of Clinical Sciences, College of Pharmacy and Health Sciences, Ajman University , Ajman , UAE
3 IMU Centre for Education, IMU University , Kuala Lumpur , Malaysia
Corresponding author email: 202011154@ajmanuni.ac.ae
Keywords: insulin infusion systems, psychology, diabetes mellitus, quality of life, child

Development and validation of a theoretical domains framework-based survey instrument to investigate Malaysian community pharmacists' practices and beliefs in pre-travel consultations for prospective Hajj and Umrah pilgrims

Kadir Norazlin Abdul 1 2
Suhaimi Azyyati Mohd 3
Othman Noordin 4 5
Wahab Mohd Shahezwan Abd 2 6 7
1 Department of Pharmacy, Kuala Lumpur Health Clinic, Ministry of Health Malaysia , 53200 , Kuala Lumpur , Malaysia
2 Faculty of Pharmacy, Universiti Teknologi MARA (UiTM) Selangor Branch, Puncak Alam Campus, 42300 , Puncak Alam , Selangor , Malaysia
3 Faculty of Pharmacy, Universiti Sultan Zainal Abidin , Besut Campus, 22200 , Besut , Terengganu , Malaysia
4 Quality Use of Medicines in Umrah and Hajj Pilgrimage Research Group, Department of Pharmacy Practice, College of Pharmacy, Taibah University , 30001 , Medina , Saudi Arabia
5 School of Pharmacy, Management and Science University , 40100 , Shah Alam , Selangor , Malaysia
6 Elderly Medication and Safety, Research Interest Group, Faculty of Pharmacy, Universiti Teknologi MARA (UiTM) , Selangor Branch, Puncak Alam Campus, 42300 , Puncak Alam , Selangor , Malaysia
7 Non-Destructive Biomedical and Pharmaceutical Research Centre, Smart Manufacturing Research Institute, Universiti Teknologi MARA (UiTM) , Selangor Branch, Puncak Alam Campus, 42300 , Puncak Alam , Selangor , Malaysia
Corresponding author email: norazlin6133@gmail.com
Keywords: development and validation, theoretical domains framework, travel health, Hajj and Umrah, community pharmacy

Practices of community pharmacists in the management of minor ailments and the quality of counselling area in selected independent drugstores in Taguig, Makati, and Pateros, Philippines

De Vera-Barasi Estela
Espartero Jean Ashley B.
Carpio Marie Jae P.
Cariaga Maria Fay Nenette M.
Institute of Pharmacy, University of Makati , West Rembo , Taguig City , Philippines
Corresponding author email: estela.barasi@umak.edu.ph
Keywords: community pharmacists, management of minor ailments, community pharmacy services, standards, self-medication, quality of counselling area

Pharmacist-led community service programme using the STEADI-Rx framework to screen fall risk and enhance knowledge among middle-aged and older adults

Wahab Mohd Shahezwan Abd 1
Ali Aida Azlina 2
Zulkifli Muhammad Harith 2
Azlan Saliha 2
Mustaffa Mohd Faiz 2
Ismail Farhana Fakhira 1
1 Department of Pharmacy Practice and Clinical Pharmacy, Faculty of Pharmacy, Universiti Teknologi MARA (UiTM) , Selangor , Malaysia
2 Department of Pharmacology and Life Sciences, Faculty of Pharmacy, UiTM , Selangor , Malaysia
Corresponding author email: mohdsh2790@uitm.edu.my
Keywords: pharmacist-led intervention, fall prevention, STEADI-Rx framework, fall-risk-increasing drugs, older adults, community-based education

Outcomes and perceptions of pharmacist-delivered Common Illness services in community pharmacies

Srimongkhol Piangkwan 1
Rachada Pattharaporn 1
Leenalard Pichaya 1
Phimarn Wiraphol 2
Potisarach Pemmarin 1
1 International Primary Care Practice Research Unit (iPCPRU), Faculty of Pharmacy, Mahasarakham University , Khamriang Sub-District, Kantharawichai District , Maha Sarakham , 44150 , Thailand
2 Social Pharmacy Research Unit, Faculty of Pharmacy, Mahasarakham University , Khamriang Sub-District, Kantharawichai District , Maha Sarakham , 44150 , Thailand
Corresponding author email: pemmarin.p@msu.ac.th
Keywords: pharmacists, community pharmacy services, primary health care, patient outcome assessment, Thailand

Factors contributing to chronic kidney disease following COVID-19 diagnosis in pre-vaccinated hospitalised patients

Khan Kashif Ullah 1
Ramatillah Diana Laila 2
1 University Malaya , Malaysia
2 Faculty of Pharmacy, Universitas 17 Agustus 1945 Jakarta, Jakarta, Indonesia
Corresponding author email: diana.ramatillah@uta45jakarta.ac.id
Keywords: COVID-19, chronic kidney disease, risk factors, survival rate

Analysis of the quality of life of patients with drug-resistant tuberculosis (DR-TB) using the EQ-5D-5L instrument at Persahabatan General Hospital, Jakarta

Manninda Reise 1
Widianti Chandra 2
Anggriani Yusi 1
Khairani Sondang 1
Andayani Nurita 1
Pratita Rasta Naya 1
Jemmima Jeanice 1
1 Universitas Pancasila , Jakarta , Indonesia
2 Persahabatan Hospital , Jakarta , Indonesia
Corresponding author email: reisemanninda@univpancasila.ac.id
Keywords: drug-resistant tuberculosis (DR-TB), quality of life (QoL), EQ-5D-5L, sociodemographic factors

Statins for primary prevention of venous thromboembolism in cancer patients: a systematic review and meta-analysis

Aomsin Narakorn 1
Prasertworakul Chatchon 2
1 Ratchaphiphat Hospital , Thailand
2 Thai CaseMix Centre (TCMC) , Thailand
Corresponding author email: narakorn_aomsin@hotmail.com
Keywords: venous thromboembolism, neoplasms, statin, primary prevention, meta-analysis

Development of a chatbot for educating patients and monitoring dermatologic conditions using topical corticosteroids

Chaipichit Natapohn 1
Ariyabaraneekul Sanruthai 1
Warangphongsi Patchamon 1
Kitikannakorn Nantawarn 1
Rujiwetpongstorn Rujira 2
1 Department of Pharmaceutical Care, Faculty of Pharmacy, Chiang Mai University , Thailand
2 Division of Dermatology, Department of Internal Medicine, Faculty of Medicine, Chiang Mai University , Thailand
Corresponding author email: natapohn.ch@cmu.ac.th
Keywords: chatbot, dermatologic diseases, topical corticosteroids, patient education, treatment monitoring

Jumpasri Project: advancing community herbal agriculture for high-potency health product development in Thailand

Ploylearmsang Chanuttha 1
Kumrae Namthip 2
Senakun Chadaporn 3
Appamaraka Sombat 3
Rattarom Ruchiluk 1
Wongsaphan Montree 4
Nualkaew Somsak 1
Yatniyom Ongart 4
1 Faculty of Pharmacy, Mahasarakham University , Thailand
2 Faculty of Environment and Resource Science, Mahasarakham University , Thailand
3 Walai Rukhavej Botanical Research Institute, Mahasarakham University , Thailand
4 Faculty of Education, Mahasarakham University , Thailand
Corresponding author email: chanuttha.p@msu.ac.th
Keywords: plants, medicinal, agriculture, community participation, pharmacists

Attitude and practices related to the implication of artificial intelligence in research among post-graduate pharmacy students of Pakistan

Rahim Najia 1
Nesar Shagufta 2
Jamshed Shazia 3
1 Dow University of Health Sciences , Pakistan
2 Jinnah College of Pharmacy, Sohail University , Karachi , Pakistan
3 Department of Pharmacy Practice, School of Pharmacy, International Medical University , Kuala Lumpur , Malaysia
Corresponding author email: najia.rahim@duhs.edu.pk
Keywords: artificial intelligence, education, pharmacy, graduate, attitude of health personnel, ethics

The acceptance of biojustice – antibiotics justification (BioJ-Abx) webApp in antibiotics prescribing among medical officers

Chan Laura Pei Yian 1
Chua Chee Lim 2
1 Samarahan District Health Office , Samarahan , Sarawak , Malaysia
2 Asajaya Health Clinic , Samarahan , Sarawak , Malaysia
Corresponding author email: laurachan@moh.gov.my
Keywords: anti-bacterial agents, artificial intelligence, primary health care, clinical decision support systems

Strengthening clinical pharmacy practice in LMICs: development and validation of a competency framework and EPA-based assessment tool in Lao PDR

Sibounheuang Vanlounni 1
Chaiyasong Surasak 2
Anusornsangiam Wanarat 2
1 Faculty of Pharmacy, University of Health and Science , Laos
2 Faculty of Pharmacy, Mahasarakham University , Thailand
Corresponding author email: surasak.c@msu.ac.th
Keywords: clinical pharmacy competency, competency framework, Entrustable Professional Activities-based (EPA) assessment tool, Lao PDR

Mediminder: a smart device for enhancing medication adherence of selected maintenance medication users in a local university in NCR, Philippines

Aquino Joshua Jae F. 1
Jaojoco Alianah Isabelle 1
Mesa Rhezlyn Joy R. 1
Aquino Jhoriz Rodel F. 2
Ocampo Justine Marie 1
Cariaga Maria Fay Nenette 1
De Vera-Barasi Estela 1
1 University of Makati , Metro Manila , Philippines
2 Independent Researcher
Corresponding author email: jaquino.a12240645@umak.edu.ph
Keywords: medication adherence, smart medication adherence products (SMAPs), digital health, chronic disease management, SDG 3, SDG 9

Living with asthma in Malaysia: patients' voices on knowledge, challenges, and support

Alyas Saba 1
Hussain Rabia 1
Ababneh Bayan Faisal 1
Muneswarao Jaya 2
1 School of Pharmaceutical Sciences, Universiti Sains Malaysia , Malaysia
2 Pharmacy Department, Hospital Pulau Pinang, Ministry of Health Malaysia , Malaysia
Corresponding author email: rabia.hussain@usm.my
Keywords: asthma self-management, knowledge, perceptions, facilitators, barriers, qualitative study

Preparedness of BS pharmacy students on industrial pharmacy internship

Alcantara Fatima Claire
Atienza Sean Thomas
Deocampo Daphne Aerin
Egipto Judy Althea
Pangilinan Carlo
Luna Milette
National University Manila , Philippines
Corresponding author email: mlteodosio@national-u.edu.ph
Keywords: preparedness, industrial pharmacy, pharmacy education, regulatory affairs, internship

Direct reporting of adverse drug reactions (ADRs): knowledge, attitude, and practice among consumers

Alcantara Vina Karyll
Orara Alistair
Joyce Honny
Belir Ma
Peralta Ana Princess
Pagunsan Maria Wiebke
Cruz-Yap Sharmaine Dela
Sabado Anna Andrea
National University Manila , Philippines
Corresponding author email: vinacalderon3312@gmail.com
Keywords: pharmacovigilance, ADR, direct ADR reporting, FDA, consumers

Effects of thiamine in patients with sepsis and septic shock: a retrospective cohort study

Leelathanalerk Areerut 1
Sripong Peeraya 1
Yangtisan Kullasinee 2
Sakulchonsin Wirata 2
Chaiyasong Chutimaporn 3
1 Health Services and Pharmacy Practice Research and Innovation, Faculty of Pharmacy, Mahasarakham University , Thailand
2 Faculty of Pharmacy, Mahasarakham University , Thailand
3 Mahasarakham Hospital , Maha Sarakham 44000
Corresponding author email: areerut.l@msu.ac.th
Keywords: thiamine, sepsis, septic shock, mortality

Limited roles, strong support: reasons for lack of pharmacist involvement in TB-DOTS facilities in Quezon City

Lalimarmo Siarra Louise T.
Tambis Joseph Stephen T.
Cu Mikee Allysa M.
Dichoso Michaela Angela P.
Matan Glaiza T.
Trinity University of Asia , Philippines
Corresponding author email: precioussiarra.1204@gmail.com
Keywords: pharmacists, tuberculosis, healthcare, counselling, barriers, public health

Evaluation of the policy implementation effectiveness of China's ‘List of Encouraged Pediatric Drugs for Research, Development, and Application’

Liu Xingchen
Yang Caijun
Wei Haoqi
Liu Xingyu
Liu Xiaoyong
Zhang Jinwei
Xi’an Jiaotong University , China
Corresponding author email: yangcj@xjtu.edu.cn
Keywords: children's medication, policy, dosage forms, effectiveness of implementation, encouragement of research and development

Regulatory and operational challenges in Thailand's narcotic and psychotropic drug supply chain: a qualitative analysis

Chanto Sanchai 1
Sooksriwong Chaoncin 1
Wasusri Thananya 2
1 Faculty of Pharmacy, Thammasat University , Rangsit Campus , Pathum Thani , Thailand
2 Faculty of Engineering, Mahidol University , Nakhon Pathom , Thailand
Corresponding author email: chaoncin@tu.ac.th
Keywords: narcotics, supply and distribution, psychotropic drugs, drug and narcotic control, pharmaceutical services

An evaluation of digitalisation implementation and risk management in the application of Good Distribution Practices (GDP) in pharmaceutical distributor: a systematic literature review 2019–2024

Tondok Eka Trisia Rante
Prima Sylvia Rizky
Pharmacy Faculty, Universitas 17 Agustus 1945 Jakarta , Jakarta, Indonesia
Corresponding author email: rantetondok@outlook.com
Keywords: digitalisation, risk management, Good Distribution Practices (GDP), pharmaceutical distribution, digital quality management system, Internet of Things (IoT)

Impact assessment of the Philippine Association of Colleges of Pharmacy (PACOP) Pharmacy Refresher Programme on completers, lecturers, and schools

Luna Arjim 1 2
Gambalan Nimfa 2
Cruz Vieno Gino 1
Penaloza Roland Amiel 1
Gulmatico Maureen Allysandra 1
Adamson Mark Harvey 1
Cariaga Maria Fay Nenette 3
Miranda Shiela 4
1 Philippine Women’s University , Philippines
2 National University , Philippines
3 University of Makati , Philippines
4 Virgen Milagrosa University Foundation , Philippines
Corresponding author email: lunaarjim@gmail.com
Keywords: Philippine Association of Colleges of Pharmacy, Pharmacists Licensure Examination, PACOP Refresher Programme

Antibacterial potential of curcumin against antibiotic-resistant bacteria: a systematic review of literature

Lengkey Veisy Dianty
Ramatillah Diana Laila
Universitas 17 Agustus 1945 Jakarta , Jakarta , Indonesia
Corresponding author email: veisydiantylengkey@gmail.com
Keywords: curcumin, antibacterial activity, antibiotic resistance, MRSA, nanoparticles

Future direction on pharmacy research and practice

Babar Zaheer-Ud-Din
College of Pharmacy, QU Health, Qatar University , Doha , Qatar
Corresponding author email: z.babar@qu.edu.qa
Keywords: pharmacy practice, future, research

